# Assessing the Early Economic Feasibility of a Curative Gene Therapy for Multiple Sclerosis Using a Risk-Adjusted Valuation Framework

**DOI:** 10.3390/healthcare14050674

**Published:** 2026-03-06

**Authors:** Attila Imre, Balázs Nagy, Rok Hren

**Affiliations:** 1 Center for Health Technology Assessment, Semmelweis University, 1085 Budapest, Hungarybalazs.nagy@syreon.eu (B.N.); 2 Center for Pharmacology and Drug Research & Development, Semmelweis University, 1085 Budapest, Hungary; 3 Syreon Research Institute, 1142 Budapest, Hungary; 4 Institute of Mathematics, Physics, and Mechanics, 1000 Ljubljana, Slovenia; 5 School of Engineering and Management, University of Nova Gorica, 5000 Nova Gorica, Slovenia

**Keywords:** tolerogenic gene therapy, multiple sclerosis, early-stage health technology assessment, risk-adjusted net present value, cost-effectiveness, advanced therapy development

## Abstract

**Background/Objectives**: Multiple sclerosis (MS) imposes a substantial clinical, humanistic, and economic burden, and current disease-modifying therapies require lifelong administration without restoring immune tolerance. IMMUTOL, a tolerogenic gene therapy under development within an EU-funded programme, aims to induce durable remission. **Methods**: This study assessed the early financial feasibility of IMMUTOL using a structured risk-adjusted net present value (rNPV) model, incorporating development and operating costs, probabilities of clinical and regulatory success, manufacturing expenditure, market dynamics, and revenue projections. Uncertainty was examined through one-way, probabilistic, and scenario analyses. **Results**: Under base-case assumptions, IMMUTOL generated a deterministic rNPV of −$223.8 million with an internal rate of return of 3.4%. Probabilistic analysis yielded a mean rNPV of −$99.4 million and a mean internal rate of return of 10.5%, with 70.2% of simulations producing negative values. Only scenarios combining higher treatment prices with lower manufacturing costs produced consistently positive rNPVs; a price of $1.5 million with a $200,000 production cost resulted in an rNPV of $711.2 million and an internal rate of return of 20.7%. Neither increased market size, reduced time to approval, nor modest cost reductions altered the conclusion. **Conclusions**: These findings emphasise a structural gap between value-based pricing and the pricing required for commercial viability. Without external support or reductions in cost structures, commercial development may be economically unattractive.

## 1. Introduction

Multiple sclerosis (MS) is a chronic and progressive immune-mediated neurological disorder that creates a substantial clinical [1,2,3,4,5,6,7,8,9,10,11,12], humanistic [13,14,15,16,17,18], and economic burden [19,20,21,22,23,24,25,26,27,28,29,30,31,32,33,34,35,36,37,38]. Current disease-modifying therapies reduce relapse frequency and delay progression, but they must be administered continually because they do not restore immune tolerance. Their long-term use involves adverse events, adherence challenges, and considerable cumulative cost. In response to these limitations, IMMUTOL, an EU-funded project under Horizon Europe (Grant 101080562), is developing gene therapy to re-establish immune tolerance via genetically enhanced vitamin D3–modified tolerogenic dendritic cells [39,40,41,42,43]. These emerging therapies hold the potential to induce long-lasting remission or even a functional cure in selected populations of patients with MS.

The development of such advanced medical technologies requires long timelines and major investment. Recent analyses show that a complete development cycle from concept to market introduction often requires about 10 years and about 2 billion dollars [44,45,46]. Early economic evaluation is, therefore, essential. Early-stage health technology assessment (eHTA) provides a structured method to estimate the potential price and the risk-adjusted net present value (rNPV) of an investigational therapy. Without explicit valuation models, innovators cannot form a coherent commercial plan and promising technologies may not reach patients [47,48,49,50].

Prior work by Imre et al. (2025) [50] presented an eHTA assessment of IMMUTOL, a novel gene therapy under development for MS, evaluating its potential long-term clinical and economic value using the ErasmusMC/iMTA MS microsimulation model [50,51,52,53,54,55,56]. By comparing IMMUTOL to a broad range of high-efficacy and escalation-based disease-modifying therapy sequences, the analysis shows that IMMUTOL could be cost-effective, and even dominant, at value-based price points up to €200,000, and remained within accepted Dutch willingness-to-pay thresholds at a one-time price of €500,000 under plausible clinical efficacy assumptions. Model scenarios have indicated that substantial reductions in relapse rates and disability progression (IRR ≤ 0.2, RR ≤ 0.1) are required for the therapy to maintain favourable cost-effectiveness [50].

These findings, supported by probabilistic sensitivity analyses showing a high probability of cost-effectiveness, have provided early strategic insight into the pricing, development pathway, and potential health-system impact of curative gene therapies for MS. In fact, health economists at established pharmaceutical companies routinely undertake eHTA analyses to judge the future value of internal development projects and acquisitions. However, due to the confidential nature of such analyses, these models are seldom published [47,48].

The BIO-QLS-Informa analysis of clinical development success rates (2011–2020) has provided a comprehensive evaluation of drug development outcomes, covering 12,728 phase transitions across 9704 programs and 1779 companies. The study reported an overall likelihood of approval (LOA) from Phase I of just 7.9%, confirming the persistent challenge of translating early-stage programs into approved therapies. Phase II, in which proof of efficacy and tolerability need to converge, remained the dominant bottleneck, with only 28.9% of candidates advancing, while disease area, drug modality, biomarker-guided enrolment, and prior target validation emerged as key determinants of success. Notably, for haematology, rare diseases, and advanced modalities—such as chimeric antigen receptor T-cell (CAR-T) and messenger ribonucleic acid (mRNA) therapeutics—demonstrated substantially higher LOAs, whereas solid tumour oncology and prevalent chronic diseases continued to show low success rates. Apparently, the success rate is higher in the development of gene therapies, which may benefit from accelerated regulatory pathways, improved delivery technologies, and clustered regularly interspaced short palindromic repeat (CRISPR)-based precision editing, though still facing challenges related to manufacturing complexity and long-term safety oversight [57].

Sancho-Martinez et al. (2025) [58] argued that traditional corporate finance methods fail to adequately value development-stage biotech companies because such firms lack predictable cash flows and instead derive value from uncertain, future R&D outcomes. The author proposed a layered valuation framework combining rNPV, Monte Carlo simulations, and Real Options Analysis to more accurately reflect the high attrition risk, long time horizons, and regulatory uncertainties endemic to biotech [58].

On an operational level, a structured rNPV presents a pharmaceutical industry standard, with analysis guiding several aspects of commercial planning. It informs the decision to continue or discontinue a project (go/no-go decision), supports the selection of patient groups and indications (market segmentation), and provides a basis for a pricing and payment strategy across indications and regions (payment model). It also arranges the sequence of development and commercial actions that lead to launch or exit. Throughout development, as evidence accumulates, the analysis can be updated.

Although our prior early-stage health technology assessment demonstrated that IMMUTOL could be cost-effective at plausible value-based price points [50], cost-effectiveness alone does not determine whether a development programme is financially viable. Gene therapy development is characterised by high upfront R&D expenditure, long timelines, substantial technical and regulatory risk, and complex manufacturing requirements. Even therapies that generate favourable incremental cost-effectiveness ratios may fail to attract investment if expected returns do not compensate for development risk and capital costs. This tension is particularly relevant in high-prevalence therapeutic areas, such as MS, where competitive standards of care raise the evidentiary requirements and increase development costs. Therefore, a structured financial evaluation that explicitly incorporates probabilities of success, timing of cash flows, discounting, and market dynamics is necessary to determine whether commercial development is economically feasible. The purpose of the present research is to construct such a structured rNPV model for the IMMUTOL gene therapy and to assess its early economic feasibility, assuming curative efficacy in MS.

## 2. Materials and Methods

**Figure 1:**
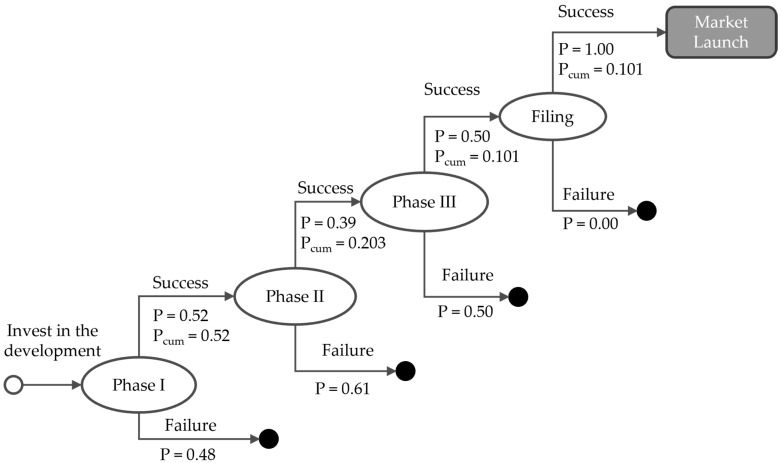
Schematic diagram of the risk-adjusted net present value model. During the development period (0–96 months), there are four decision points; to each, a probability of success or failure is assigned. The cumulative probability of approval is 10.1%.

We used an rNPV framework to quantify the expected financial value of the IMMUTOL development programme [59,60,61,62,63]. The rNPV model was constructed in discrete monthly time steps and included the sequence of development costs, post-approval commercial activities, expected revenues, and expected manufacturing and operating expenditures. All financial quantities are expressed as expected values because they depend on the probability that the product progressed successfully through the clinical and regulatory pathway. Figure 1 presents a schematic diagram of the risk-adjusted model during the development phase. After the investment decision is made, there are four decision nodes where there is a probability of success or failure. Also assigned to these points are the necessary costs of development, i.e., funding the trials at different stages and funding market access activities. The cumulative probability of success from the investment decision to market entry is 10.1%.

Expected net cash flow in month t was denoted by $CF_{t}$. Cash flows were discounted to present value using a constant annual discount rate r with monthly compounding. The risk-adjusted net present value was defined as

(1) $$rNPV=\sum_{t=0}^{T}\frac{CF_{T}}{{\left(1+r\right)}^{t/12}}$$

This formulation extended a standard discounted cash flow approach by incorporating explicit probabilities of success for development and regulatory progression. All model parameters (including base-case values and ranges for sensitivity analysis) are detailed in Appendix A.

### 2.1. Modelling of Market Share

The market share model describes how the share of a new therapy changes over time in a way that reflects diffusion during early adoption and decline as competing products enter the market. The aim is to represent this trajectory with a simple and interpretable function that remains bounded and continuous. Time t is measured in years after launch, and market share is written as function $\;f\left(t\right)$.

The model defines four parameters that determine the overall shape. The initial share $s_{0}$ specifies the market share at launch, M denotes peak market share. The time to peak market share $T_{max}$ specifies the month in which that maximum occurs. The terminal share $s_{\infty }$ specifies the long run level after decline. Two rate parameters control the steepness of the curve. The growth rate $k_{1}$ governs how quickly the product approaches its peak, and the decline rate $k_{2}$ governs how quickly the product falls from its peak toward its terminal level.

The model expresses the growth phase with an exponential saturation function that rises from $s_{0}$ to M and reaches M exactly at $T_{max}$. For all $t\leq T_{max}$ the function is:

(2) $$f\left(t\right)=s_{0}+\left(M-s_{0}\right)\frac{1-e^{-k_{1}t}}{1-e^{-k_{1}T_{max}}}$$

The model expresses the decline phase with an exponential decay function that begins at M and converges to $s_{\infty }$. For all $t>T_{max}$, the function is:

(3) $$f\left(t\right)=s_{\infty }+\left(M-s_{\infty }\right)e^{-k_{2}(t-T_{max})}$$

These two equations together produce a continuous function that increases, peaks, and then declines toward a stable level. The parameters can be estimated from empirical data or chosen to match expected adoption patterns, and the resulting function can be used directly in the economic evaluation.

### 2.2. Uncertainty Analysis

Uncertainty analysis used both one-way sensitivity analysis (OWSA) and probabilistic sensitivity analysis (PSA). In the OWSA, each parameter was varied independently by 10% above and below its base-case value, and the resulting changes in expected net present value were recorded. The ten parameters with the largest absolute effect were summarised in a tornado diagram.

The PSA examined the combined effect of parameter uncertainty. Each uncertain input was assigned a probability distribution, and Monte Carlo sampling drew one value from each distribution for every iteration. These sampled values were passed through the full model to generate a distribution of expected net present values. The PSA used 1000 iterations, which were sufficient to obtain stable estimates of uncertainty in the outputs.

### 2.3. Scenario Analysis

Scenario analysis evaluated the effect of alternative structural and economic assumptions on projected financial outcomes. Each scenario replaced one or more base-case assumptions with a predefined alternative, and the model was re-run without further changes to inputs or structure. The analysis focused on assumptions judged to be influential for long-term value, including the discount rate, manufacturing costs, market share trajectory, time to marketing authorisation, and combinations of drug price and production cost.

We also assessed a scenario in which the model was not risk-adjusted, meaning all probabilities of success for successive phases were set to 100%. In conjunction with this, because the model was no longer risk-adjusted, higher discount rates between 20% and 30% were applied. For each scenario, the model produced a net present value, an internal rate of return, a payback period when applicable, peak annual sales, and peak annual patient numbers. The decision criterion remained the same across all scenarios: a net present value above zero indicated a go decision, and a net present value at or below zero indicated a no-go decision.

## 3. Results

Under base-case assumptions (detailed in Appendix A), the IMMUTOL programme generated a negative financial outcome. The deterministic rNPV was −$223.8 million, with a 3.4% internal rate of return. The probabilistic analysis (1000 iterations) produced a mean rNPV of −$99.4 million and a 10.5% mean internal rate of return. The difference is the consequence of a positively skewed distribution of NPVs. Most iterations (72.9%) produced negative values, while a smaller set of draws combining favourable economic and technical parameters yielded high positive outliers, with some rNPV values above $2 billion.

**Figure 2:**
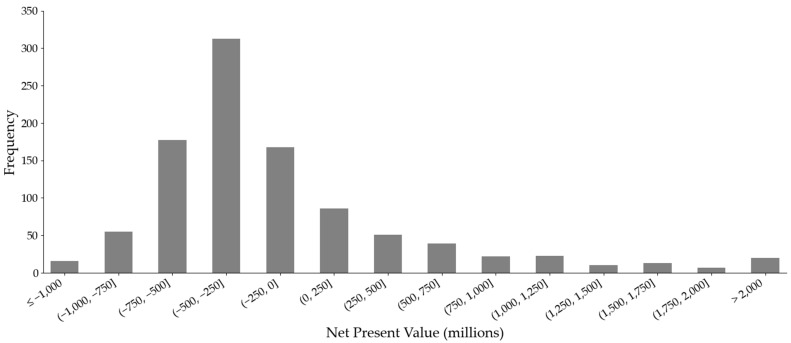
Histogram of probabilistic net present value calculations.

**Figure 3:**
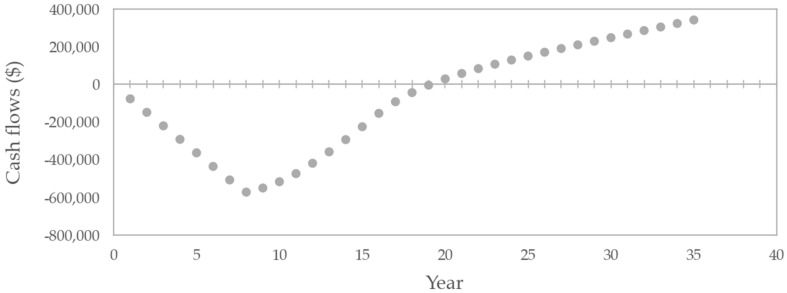
Cumulative undiscounted cash flows in the base-case analysis.

**Figure 4:**
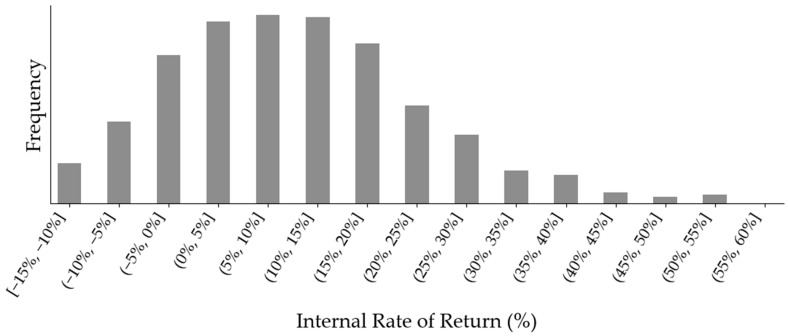
Histogram of probabilistic internal rate of return calculations.

Across all Monte Carlo iterations, 729 of 1000 (72.9%) resulted in rNPV values below zero (Figure 2). The distribution concentrated in the negative region, although a long, right-hand tail was present. The project did not recover its investment within the model horizon under base-case discounting. The cumulative undiscounted cash flow curve in Figure 3 shows that total cash flows became positive only after year 19 from the model’s start. Peak annual sales reached $217.5 million, corresponding to 4730 patients in the peak year. Figure 4 presents a histogram of the probabilistic internal rate of return calculations. Overall, 43.8% of iterations produced a positive internal rate of return.

**Figure 5:**
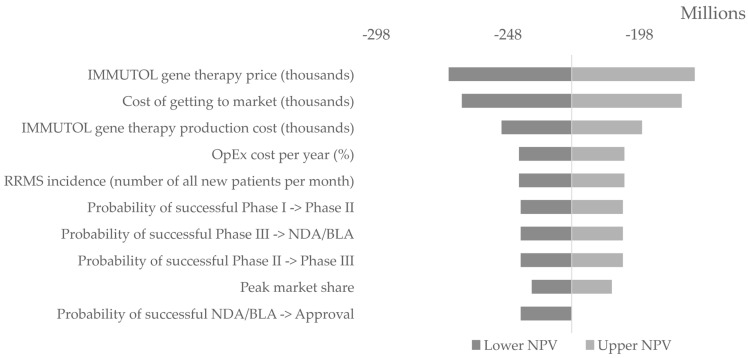
Tornado plot of the most sensitive parameters.

The OWSA varied each parameter by 10% around its base-case value. Treatment price was the most influential driver, with an rNPV swing of $93.5 million across its range. The total cost of getting to marketing authorisation generated the next largest effect of $83.6 million. Production cost per treatment produced a $53.4 million change. RRMS incidence and annual operating expenditure each shifted rNPV by $40.1 million. The three probabilities of successful transition between clinical phases each generated a $38.8 million difference. Peak market share produced a $30.4 million swing. Figure 5 displays the tornado plot ranking and magnitude of these effects.

Scenario analysis examined structural changes to key assumptions (Table 1). Removing discounting generated a positive NPV of $353.5 million and produced a payback period of 231 months (19.3 years). Increasing the discount rate yielded negative outcomes across all tested rates. Higher manufacturing costs resulted in negative rNPV values, and expanding the peak patient population did not generate a positive result. A shortened time to marketing authorisation did not improve the rNPV. Combinations of higher therapy prices with lower production costs were the only scenarios that produced consistently positive rNPV values. The most favourable tested combination, a price of $1.5 million and a production cost of $200,000, produced an rNPV of $711.2 million.

The scenarios without risk adjustment evaluated cases in which all probabilities of success were set to 100% and discount rates were increased to 20–30%. At a 30% discount rate, the model produced an NPV of –$51.1 million. At 25% and 20% discount rates, NPVs were $55.3 million and $259.2 million, respectively. Under these conditions, payback periods ranged from 159 to 191 months, and peak annual sales increased to $2.17 billion.

## 4. Discussion

This analysis evaluated the financial viability of developing the IMMUTOL tolerogenic gene therapy using an rNPV framework. Under base-case assumptions, the programme did not generate positive value. The deterministic rNPV was negative, and the probabilistic analysis showed that 70.2% of simulations also produced negative values. The internal rate of return remained below levels typically sought by private investors in risky biotechnology development. Taken together, the results indicate that, given current expectations about costs and risks, the programme is unlikely to be financially attractive.

A central finding is the gap between a value-based price and the price required for commercial viability. Previous work estimated a value-based price below approximately $500,000. In contrast, this model produced positive rNPV only when the treatment price exceeded about $1 million and manufacturing cost fell to, or below, $200,000. This difference cannot be closed through plausible changes in uptake, eligible population, or incremental reductions in production cost. The implication is that the price that reflects health-system value is substantially lower than the price needed to attract private investment, and this gap limits the feasibility of commercial development [64,65,66,67,68,69,70,71,72,73].

A broader policy context may help interpret these findings. Many health systems are grappling with how to reimburse one-time, high-cost curative therapies while maintaining budget sustainability [74,75,76,77,78,79,80,81,82]. Current reimbursement frameworks, largely based on upfront payment, may not align with the risk profile or societal value of tolerogenic gene therapies. Alternative payment models (e.g., outcomes-based annuities [83], risk-sharing schemes, or publicly supported development funds) could narrow the gap between value-based prices and required commercial prices; however, these mechanisms remain limited in scope, inconsistently applied across jurisdictions. While this analysis does not prescribe policy responses, it indicates that, without external intervention or changes in development costs, economic incentives may be insufficient to support continued development.

The model’s second most influential parameter was the cost of development through market approval. Although a figure of approximately $600 million aligns with published estimates, an argument could be made that tolerogenic gene therapies might achieve approval with smaller trial programmes. However, for a high-prevalence disease where multiple treatments are available, such as multiple sclerosis, regulators would likely require larger, comparative Phase III trials powered to detect clinically statistically significant differences against the current standard of care. Power calculations under these conditions yield enrolment numbers far higher than those of typical gene therapy studies. Consequently, even though gene therapies are sometimes approved on limited datasets for ultra-rare conditions, such precedents do not readily generalise to a large, competitive therapeutic area. This would limit the extent to which development costs could realistically fall, even under optimistic assumptions.

Due to these findings, several alternative development strategies merit consideration. One option is to focus on rare indications with high unmet need and limited (or no) existing treatments. These settings often permit smaller trials, shorter follow-up, and higher economically justifiable prices. The early modelling exercise for relapsing–remitting MS remains informative in this context. Without an explicit financial model for a large indication, it would not be clear that a niche indication offers a more credible financial path. The finding that the IMMUTOL gene therapy is unlikely to be viable in a mainstream indication, therefore, clarifies why a “niche-buster” approach may be appropriate [84,85,86].

A second strategy is to sequence patients by concentrating development in subgroups with limited response to current therapies. Treatment-resistant or rapidly progressing forms of MS fit this profile. A related method is staged expansion, in which development begins in a refractory subgroup or in a population identified by validated biomarkers. Broader phenotypes follow once safety and activity are established. These staged programmes could meet regulatory expectations with smaller studies for early label expansions, which reduces financial risk.

Other strategies may aim to share financial risk. Partnerships or licensing agreements during development can transfer substantial downstream costs to a partner with the capacity to complete late-stage trials and commercialisation. These arrangements are common in advanced therapy development and can temper the late-stage risks (and associated negative NPV) in the present model.

The present analysis shows the value of early health economic modelling in the development of investigational medical products, specifically gene therapy. The same approach can be applied to other pharmaceuticals, medical devices, and investigational diagnostic modalities [87,88,89,90,91,92]. Early modelling can identify development directions that are unlikely to be economically viable, and can help redirect strategies towards justifiable directions. In this role, the model functions as a decision-supporting tool that guides whether development should continue and in what form.

Several non-commercial development pathways also merit consideration. Public–private partnerships and nonprofit development structures have supported vaccine development and research in neglected diseases [85,86,93]. These arrangements can sustain programmes that have substantial clinical value but limited commercial appeal. The results of this analysis suggest that such pathways may be relevant for tolerogenic therapy in multiple sclerosis.

The study applies eHTA methods to a publicly funded research programme, uses fully disclosed parameter inputs, and reports the mathematical specification of its market-share function. It includes one-way, probabilistic, and scenario-based sensitivity analyses, allowing a systematic assessment of parameter uncertainty; these elements improve transparency and reproducibility.

However, the analysis also has limitations. Many inputs rely on the literature estimates or expert judgement, which introduces uncertainty that could only partially be quantified. Manufacturing costs, remission rates, and long-run market dynamics for tolerogenic gene therapies remain uncertain at this stage of development. The competitive landscape over a decade or longer could change substantially from current assumptions. The model, therefore, provides an early estimate that requires revision as information and empirical evidence accumulate. Future research would benefit from greater transparency in reporting NPV models for advanced therapies. These models are widely used in industry but are rarely published, which limits methodological development and comparative assessment. A more complete collection of the academic literature would support public funding decisions, improve comparability across studies, and clarify the extent to which financial feasibility aligns with clinical or societal value.

This study has identified economic constraints that limit the feasibility of commercial development. These constraints do not imply a lack of clinical promise. They indicate that current structures produce a gap between social value and financial return. As programme-specific clinical data become available, updating the model will allow a more accurate assessment of feasibility and inform decisions about whether to proceed with development. Another aspect that has, until recently, been largely overlooked is the role of patient and public involvement in health technology assessment [94,95]. Integrating patient perspectives more effectively across the development process can strengthen methodological quality, ensure alignment with patient needs, and enhance the broader societal relevance of the work.

$$rNPV=\sum_{t=0}^{T}\frac{CF_{T}}{{\left(1+r\right)}^{t/12}}f\left(t\right)=s_{0}+\left(M-s_{0}\right)\frac{1-e^{-k_{1}t}}{1-e^{-k_{1}T_{max}}}f\left(t\right)=s_{\infty }+\left(M-s_{\infty }\right)e^{-k_{2}(t-T_{max})}$$

## 5. Conclusions

The results indicate that profitability of investing in the development of the IMMUTOL therapy strongly depends on price and manufacturing cost, rather than on time to market or patient reach. Although a lower price of IMMUTOL could improve access, there is a high probability that it would not be economically sustainable. The combination of factors, such as high production costs inherent to gene therapy manufacturing and a crowded drug market with already effective therapies, can limit profitability even under optimistic, large-scale uptake scenarios.

## Figures and Tables

**Table 1:** Base-case results and scenario analysis.

Scenario	Net Present Value	Internal Rate of Return	Payback Period (Months)	Peak Sales (Trailing 12 Months)	Peak Patient Number (Trailing 12 Months)	Decision: Go/No-Go
***Base***						
Deterministic	−223,759,369	3.5%	-	$217,450,828	4730	No-Go
Probabilistic	−99,388,267	10.5%	-	$273,367,639	7460	No-Go
***Discounting***						
Discount rate—0%	353,506,944	3.4%	231	$217,450,828	4730	Go
Discount rate—5%	−86,566,876	3.4%	-	$217,450,828	4730	No-Go
Discount rate—15%	−262,907,323	3.4%	-	$217,450,828	4730	No-Go
Discount rate—20%	−267,114,087	3.4%	-	$217,450,828	4730	No-Go
Discount rate—25%	−258,569,622	3.4%	-	$217,450,828	4730	No-Go
***Cost of goods sold***						
500 k drug cost, 100 k production cost	−90,198,737	7.8%	-	$217,450,828	4730	No-Go
***Market share***						
Peak patients 10,000 per year	−58,911,557	8.5%	-	$451,962,225	9830	No-Go
Marketing authorisation in 6 years	−218,569,380	3.9%	-	$217,450,828	4730	No-Go
***Drug price & production cost***						
Drug price 1 M, 200 k production cost	243,702,841	14.6%	186	$434,901,656	4730	Go
Drug price 1 M, 400 k production cost	−23,418,422	9.5%	-	$434,901,656	4730	No-Go
Drug price 1 M, 600 k production cost	−290,539,684	0.4%	-	$434,901,656	4730	No-Go
Drug price 1.5 M, 200 k production cost	711,165,051	20.7%	151	$652,352,484	4730	Go
Drug price 1.5 M, 400 k production cost	444,043,788	19.2%	157	$652,352,484	4730	Go
Drug price 1.5 M, 600 k production cost	176,922,525	17.6%	166	$652,352,484	4730	Go
***Risk-adjustment analysis***						
Likelihood of approval 100%, Discount—30%	−51,093,356	27.2%	-	$2,170,882,905	4730	No-Go
Likelihood of approval 100%, Discount—25%	55,285,295	27.2%	191	$2,170,882,905	4730	Go
Likelihood of approval 100%, Discount—20%	259,215,040	27.2%	159	$2,170,882,905	4730	Go

## Data Availability

The data generated in this research are available from the corresponding author upon reasonable request.

## Abbreviations

The following abbreviations are used in this manuscript:

CAR-T	Chimeric antigen receptor T-cell
CF	Cash flow
CRISPR	Clustered regularly interspaced short palindromic repeat
eHTA	Early-stage health technology assessment
LOA	Likelihood of approval
MS	Multiple sclerosis
NPV	Net present value
OWSA	One-way sensitivity analysis
PSA	Probabilistic sensitivity analysis
rNPV	Risk-adjusted net present value
RRMS	Relapsing–remitting multiple sclerosis
WTP	Willingness-to-pay

## Appendix A

**Table A1:** Input parameters.

Parameter	Base-Case Value	OWSA Range	Distribution	Reference
**General**				
Discount rate	10%	-	-	[96]
**Unit costs**				
IMMUTOL gene therapy price (thousands)	500,000	450,000–550,000	Gamma	[50]
IMMUTOL gene therapy production cost (thousands)	200,000	180,000–220,000	Gamma	Expert Opinion
Operating expenditure per month (%)	30%	27–33%	Beta	Expert Opinion
**Development variables**				
Number of months until launch	96	86–106	Normal	[97]
Cost of getting to market (thousands)	573,846,000	516,462,000–631,231,000	Gamma	[46]
Probability of successful Phase I → Phase II	52%	47–57%	Beta	[57]
Probability of successful Phase II → Phase III	39%	35–42%	Beta	[57]
Probability of successful Phase III → NDA/BLA	50%	45–55%	Beta	[57]
Probability of successful NDA/BLA → Approval	100%	90–100%	Beta	[57]
**Market share**				
RRMS incidence (number of all new patients per month)	5167	4650–5683	Normal	[98]
Market share in the 1st month	1.9%	1.7–2.1%	Beta	Expert Opinion
Peak market share	7.7%	7.0–8.5%	Beta	Expert Opinion
Time (months) to maximum market share	96	86–106	Normal	Expert Opinion
Terminal market share	2.0%	1.8–2.2%	Beta	Expert Opinion
Growth rate until maximum market share	0.02	0.018–0.022	Normal	Expert Opinion
Growth rate after maximum market share	0.03	0.027–0.033	Normal	Expert Opinion
**Launch costs**				
Post-launch marketing campaign cost	150,000,000	135,000,000–165,000,000	Gamma	[99]
Post-launch marketing campaign length in months	36	32–40	Normal	[99]

## References

[1] Claflin S.B., Tan B., Taylor B.V. (2019). The Long-Term Effects of Disease Modifying Therapies on Disability in People Living with Multiple Sclerosis: A Systematic Review and Meta-Analysis. Mult. Scler. Relat. Disord..

[2] Cruz Rivera S., Aiyegbusi O.L., Piani Meier D., Dunne A., Harlow D.E., Henke C., Kamudoni P., Calvert M.J. (2023). The Effect of Disease Modifying Therapies on Fatigue in Multiple Sclerosis. Mult. Scler. Relat. Disord..

[3] Dang Y.L., Yong V.T., Sharmin S., Perucca P., Kalincik T. (2023). Seizure Risk in Multiple Sclerosis Patients Treated with Disease-Modifying Therapy: A Systematic Review and Network Meta-Analysis. Mult. Scler. J..

[4] Elgenidy A., Abdelhalim N.N., Al-kurdi M.A., Mohamed L.A., Ghoneim M.M., Fathy A.W., Hassaan H.K., Anan A., Alomari O. (2024). Hypogammaglobulinemia and Infections in Patients with Multiple Sclerosis Treated with Anti-CD20 Treatments: A Systematic Review and Meta-Analysis of 19,139 Multiple Sclerosis Patients. Front. Neurol..

[5] Gasim M., Bernstein C.N., Graff L.A., Patten S.B., El-Gabalawy R., Sareen J., Bolton J.M., Marriott J.J., Fisk J.D., Marrie R.A. (2018). Adverse Psychiatric Effects of Disease-Modifying Therapies in Multiple Sclerosis: A Systematic Review. Mult. Scler. Relat. Disord..

[6] Gonzalez-Lorenzo M., Ridley B., Minozzi S., Del Giovane C., Peryer G., Piggott T., Foschi M., Filippini G., Tramacere I., Baldin E. (2024). Immunomodulators and Immunosuppressants for Relapsing-Remitting Multiple Sclerosis: A Network Meta-Analysis. Cochrane Database Syst. Rev..

[7] Lopez-Leon S., Geissbühler Y., Sabidó M., Turkson M., Wahlich C., Morris J.K. (2020). A Systematic Review and Meta-Analyses of Pregnancy and Fetal Outcomes in Women with Multiple Sclerosis: A Contribution from the IMI2 ConcePTION Project. J. Neurol..

[8] Mavridis T., Papagiannakis N., Breza M., Vavougios G.D., Patas K., Daponte A., Laskaratos A., Archontakis-Barakakis P., Pantazopoulos I., Mitsikostas D.D. (2022). B-Cell Targeted Therapies in Patients with Multiple Sclerosis and Incidence of Headache: A Systematic Review and Meta-Analysis. J. Pers. Med..

[9] Papadopoulos D., Gklinos P., Psarros G., Drellia K., Delicha E.M., Friede T., Mitsikostas D.D., Nicholas R.S. (2022). Disease-Modifying Treatments for Multiple Sclerosis Have Not Affected the Incidence of Neoplasms in Clinical Trials over 3 Decades: A Meta-Analysis with Meta-Regression. J. Neurol..

[10] Pipek L.Z., Mahler J.V., Nascimento R.F.V., Apóstolos-Pereira S.L., Silva G.D., Callegaro D. (2023). Cost, Efficacy, and Safety Comparison between Early Intensive and Escalating Strategies for Multiple Sclerosis: A Systematic Review and Meta-Analysis. Mult. Scler. Relat. Disord..

[11] Schubert C., Steinberg L., Peper J., Ramien C., Hellwig K., Köpke S., Solari A., Giordano A., Gold S.M., Friede T. (2023). Postpartum Relapse Risk in Multiple Sclerosis: A Systematic Review and Meta-Analysis. J. Neurol. Neurosurg. Psychiatry.

[12] Tsivgoulis G., Katsanos A.H., Grigoriadis N., Hadjigeorgiou G.M., Heliopoulos I., Kilidireas C., Voumvourakis K. (2015). The Effect of Disease Modifying Therapies on Brain Atrophy in Patients with Relapsing-Remitting Multiple Sclerosis: A Systematic Review and Meta-Analysis. PLoS ONE.

[13] Visser L.A., Louapre C., Uyl-de Groot C.A., Redekop W.K. (2020). Patient Needs and Preferences in Relapsing-Remitting Multiple Sclerosis: A Systematic Review. Mult. Scler. Relat. Disord..

[14] Hirt J., Dembowska K., Woelfle T., Axfors C., Granziera C., Kuhle J., Kappos L., Hemkens L.G., Janiaud P. (2024). Clinical Trial Evidence of Quality-of-Life Effects of Disease-Modifying Therapies for Multiple Sclerosis: A Systematic Analysis. J. Neurol..

[15] Popp R.F.J., Fierlbeck A.K., Knüttel H., König N., Rupprecht R., Weissert R., Wetter T.C. (2017). Daytime Sleepiness versus Fatigue in Patients with Multiple Sclerosis: A Systematic Review on the Epworth Sleepiness Scale as an Assessment Tool. Sleep Med. Rev..

[16] Ngorsuraches S., Poudel N. (2021). Incorporating Patients’ Preferences in the Value Assessment of Disease-Modifying Therapies for Multiple Sclerosis: A Narrative Review. Expert Rev. Pharmacoecon. Outcomes Res..

[17] O’Connor A.B., Schwid S.R., Herrmann D.N., Markman J.D., Dworkin R.H. (2008). Pain Associated with Multiple Sclerosis: Systematic Review and Proposed Classification. Pain.

[18] Lee D., Newell R., Ziegler L., Topping A. (2008). Treatment of Fatigue in Multiple Sclerosis: A Systematic Review of the Literature. Int. J. Nurs. Pract..

[19] Uitdehaag B., Kobelt G., Berg J., Capsa D., Dalén J. (2017). The European Multiple Sclerosis Platform New Insights into the Burden and Costs of Multiple Sclerosis in Europe: Results for the Netherlands. Mult. Scler. J..

[20] Bebo B., Cintina I., LaRocca N., Ritter L., Talente B., Hartung D., Ngorsuraches S., Wallin M., Yang G. (2022). The Economic Burden of Multiple Sclerosis in the United States: Estimate of Direct and Indirect Costs. Neurology.

[21] Simoens S. (2022). Societal Economic Burden of Multiple Sclerosis and Cost-Effectiveness of Disease-Modifying Therapies. Front. Neurol..

[22] Safiri S., Ghaffari Jolfayi A., Mousavi S.E., Nejadghaderi S.A., Sullman M.J.M., Kolahi A.-A. (2024). Global Burden of Multiple Sclerosis and Its Attributable Risk Factors, 1990–2019. Front. Neurol..

[23] Vitturi B.K., Rahmani A., Montecucco A., Dini G., Durando P. (2023). Occupational Outcomes of People with Multiple Sclerosis during the COVID-19 Pandemic: A Systematic Review with Meta-Analysis. Front. Public Health.

[24] Stawowczyk E., Malinowski K.P., Kawalec P., Moćko P. (2015). The Indirect Costs of Multiple Sclerosis: Systematic Review and Meta-Analysis. Expert Rev. Pharmacoecon. Outcomes Res..

[25] Adelman G., Rane S.G., Villa K.F. (2013). The Cost Burden of Multiple Sclerosis in the United States: A Systematic Review of the Literature. J. Med. Econ..

[26] Schauf M., Chinthapatla H., Dimri S., Li E., Hartung D.M. (2023). Economic Burden of Multiple Sclerosis in the United States: A Systematic Literature Review. J. Manag. Care Spec. Pharm..

[27] Dahham J., Rizk R., Kremer I., Evers S.M.A.A., Hiligsmann M. (2021). Economic Burden of Multiple Sclerosis in Low- and Middle-Income Countries: A Systematic Review. PharmacoEconomics.

[28] Brodszky V., Beretzky Z., Baji P., Rencz F., Péntek M., Rotar A., Tachkov K., Mayer S., Simon J., Niewada M. (2019). Cost-of-Illness Studies in Nine Central and Eastern European Countries. Eur. J. Health Econ..

[29] Mayntz S.K., Peronard C.R.F., Søgaard J., Chang A.Y. (2024). The Economic Burden of Diseases in the Nordic Countries: A Systematic Review. Scand. J. Public Health.

[30] Chataway J., Murphy N., Khurana V., Schofield H., Findlay J., Adlard N. (2021). Secondary Progressive Multiple Sclerosis: A Systematic Review of Costs and Health State Utilities. Curr. Med. Res. Opin..

[31] Kolasa K. (2013). How Much Is the Cost of Multiple Sclerosis—Systematic Literature Review. Przegl Epidemiol.

[32] Bryant J. (2001). Systematic Review of Immunomodulatory Drugs for the Treatment of People with Multiple Sclerosis: Is There Good Quality Evidence on Effectiveness and Cost?. J. Neurol. Neurosurg. Psychiatry.

[33] Diederich F., König H.-H., Mietzner C., Brettschneider C. (2018). Costs of Informal Nursing Care for Patients with Neurologic Disorders: A Systematic Review. Neurology.

[34] Lo J., Chan L., Flynn S. (2021). A Systematic Review of the Incidence, Prevalence, Costs, and Activity and Work Limitations of Amputation, Osteoarthritis, Rheumatoid Arthritis, Back Pain, Multiple Sclerosis, Spinal Cord Injury, Stroke, and Traumatic Brain Injury in the United States: A 2019 Update. Arch. Phys. Med. Rehabil..

[35] Kavaliunas A., Danylaitė Karrenbauer V., Binzer S., Hillert J. (2022). Systematic Review of the Socioeconomic Consequences in Patients with Multiple Sclerosis with Different Levels of Disability and Cognitive Function. Front. Neurol..

[36] Kavaliunas A., Danylaite Karrenbauer V., Hillert J. (2021). Socioeconomic Consequences of Multiple Sclerosis—A Systematic Literature Review. Acta Neurol. Scand..

[37] Ernstsson O., Gyllensten H., Alexanderson K., Tinghög P., Friberg E., Norlund A. (2016). Cost of Illness of Multiple Sclerosis—A Systematic Review. PLoS ONE.

[38] Paz-Zulueta M., Parás-Bravo P., Cantarero-Prieto D., Blázquez-Fernández C., Oterino-Durán A. (2020). A Literature Review of Cost-of-Illness Studies on the Economic Burden of Multiple Sclerosis. Mult. Scler. Relat. Disord..

[39] Morali K., Giacomello G., Vuono M., Gregori S. (2024). Leveraging Current Insights on IL -10-producing Dendritic Cells for Developing Effective Immunotherapeutic Approaches. FEBS Lett..

[40] Quirant-Sánchez B., Mansilla M.J., Navarro-Barriuso J., Presas-Rodríguez S., Teniente-Serra A., Fondelli F., Ramo-Tello C., Martínez-Cáceres E. (2021). Combined Therapy of Vitamin D3-Tolerogenic Dendritic Cells and Interferon-β in a Preclinical Model of Multiple Sclerosis. Biomedicines.

[41] Fondelli F., Willemyns J., Domenech-Garcia R., Mansilla M.J., Godoy-Tena G., Ferreté-Bonastre A.G., Agúndez-Moreno A., Presas-Rodriguez S., Ramo-Tello C., Ballestar E. (2024). Targeting Aryl Hydrocarbon Receptor Functionally Restores Tolerogenic Dendritic Cells Derived from Patients with Multiple Sclerosis. J. Clin. Investig..

[42] Gregori S., Amodio G., Passerini L., Santoni de Sio F.R. (2022). Alteration of Interleukin-10-Producing Type 1 Regulatory Cells in Autoimmune Diseases. Curr. Opin. Hematol..

[43] Pitter J.G., Nagy L., Nagy B., Hren R. (2024). Development Perspectives for Curative Technologies in Primary Demyelinating Disorders of the Central Nervous System with Neuromyelitis Optica Spectrum Disorder (NMOSD) and Myelin Oligodendrocyte Glycoprotein Antibody-Associated Disease (MOGAD) at the Forefront. J. Pers. Med..

[44] DiMasi J.A., Grabowski H.G., Hansen R.W. (2016). Innovation in the Pharmaceutical Industry: New Estimates of R&D Costs. J. Health Econ..

[45] Wouters O.J., McKee M., Luyten J. (2020). Estimated Research and Development Investment Needed to Bring a New Medicine to Market, 2009–2018. JAMA.

[46] Sabatini M.T., Chalmers M. (2023). The Cost of Biotech Innovation: Exploring Research and Development Costs of Cell and Gene Therapies. Pharm. Med..

[47] Kaló Z., Imre A., Tatár M., Nagy B. (2025). Early-Stage Assessment of Investigational Digital Technologies in Mental Health: A Guidance for Innovators. Psychiatry Res..

[48] Kaló Z., Imre A., Varga G., Kovács S., Tímár G., Nagy B. (2025). Optimization of Pharmaceutical Research and Development by Early-phase Assessment of Investigational Medicinal Products. Br. J. Clin. Pharmacol..

[49] Bodrogi J., Kaló Z. (2010). Principles of Pharmacoeconomics and Their Impact on Strategic Imperatives of Pharmaceutical Research and Development: Principles of Pharmacoeconomics. Br. J. Pharmacol..

[50] Imre A., Nagy B., Hren R. (2025). Early-stage Health Technology Assessment of a Curative Gene Therapy for Multiple Sclerosis. Br. J. Clin. Pharmacol..

[51] Versteegh M.M., Huygens S.A., Wokke B.W.H., Smolders J. (2022). Effectiveness and Cost-Effectiveness of 360 Disease-Modifying Treatment Escalation Sequences in Multiple Sclerosis. Value Health.

[52] Corsten C.E.A., Huygens S.A., Versteegh M.M., Wokke B.H.A., Smets I., Smolders J. (2023). Benefits of Sphingosine-1-Phosphate Receptor Modulators in Relapsing MS Estimated with a Treatment Sequence Model. Mult. Scler. Relat. Disord..

[53] Smets I., Versteegh M., Huygens S., Corsten C., Wokke B., Smolders J. (2023). Health-Economic Benefits of Anti-CD20 Treatments in Relapsing Multiple Sclerosis Estimated Using a Treatment-Sequence Model. Mult. Scler. J.-Exp. Transl. Clin..

[54] Smets I., Versteegh M., Huygens S., Wokke B., Smolders J. (2024). Benefits of Early Highly Effective versus Escalation Treatment Strategies in Relapsing Multiple Sclerosis Estimated Using a Treatment-Sequence Model. Mult. Scler. J..

[55] Versteegh M.M., Huygens S.A. (2025). Exit Strategies in Patients with Stable MS: Cost-Effectiveness of Extended Interval Dosing of Ocrelizumab and Natalizumab versus de-Escalating to Cladribine. Mult. Scler. Relat. Disord..

[56] Cloosterman S., Wijnands I., Huygens S., Wester V., Lam K.-H., Strijbis E., Den Teuling B., Versteegh M. (2021). The Potential Impact of Digital Biomarkers in Multiple Sclerosis in The Netherlands: An Early Health Technology Assessment of MS Sherpa. Brain Sci..

[57] Thomas D., Chancellor D., Micklus A., LaFever S., Hay M., Chaudhuri S., Bowden R., Lo A.W. (2021). Clinical Development Success Rates and Contributing Factors 2011–2020.

[58] Sancho-Martinez I. Architecting Value: A Framework for Biotech Asset Valuation: From rNPV to Real Options. https://inbistra.com/en/blog/biotech-valuation-framework.

[59] Stewart J.J., Allison P.N., Johnson R.S. (2001). Putting a Price on Biotechnology. Nat. Biotechnol..

[60] Svennebring A.M., Wikberg J.E. (2013). Net Present Value Approaches for Drug Discovery. SpringerPlus.

[61] Woo J., Kim E., Sung T.-E., Lee J., Shin K., Lee J. (2019). Developing an Improved Risk-Adjusted Net Present Value Technology Valuation Model for the Biopharmaceutical Industry. J. Open Innov. Technol. Mark. Complex..

[62] Bae J., Yeon J.H., Kim C. (2023). A Study on the New Risk-Adjusted Net Present Value (rNPV) Technology Valuation: About Risk-Adjustment. Int. J. Appl. Eng. Technol..

[63] Chandra A., Mazumdar S. (2024). 2024 Biotech Asset Valuation Methods: A Practitioner’s Guide. J. Invest. Manag..

[64] Garrison L.P., Jackson T., Paul D., Kenston M. (2019). Value-Based Pricing for Emerging Gene Therapies: The Economic Case for a Higher Cost-Effectiveness Threshold. J. Manag. Care Spec. Pharm..

[65] Gonçalves E. (2022). Value-Based Pricing for Advanced Therapy Medicinal Products: Emerging Affordability Solutions. Eur. J. Health Econ..

[66] Danzon P., Towse A. (2002). The Economics of Gene Therapy and of Pharmacogenetics. Value Health.

[67] Ossandon H., Armijo N., Vargas C., Repetto G.M., Espinoza M.A. (2024). Challenges for Gene Therapy in the Financial Sustainability of Health Systems: A Scoping Review. Orphanet J. Rare Dis..

[68] Garrison L.P., Lo A.W., Finkel R.S., Deverka P.A. (2023). A Review of Economic Issues for gene-targeted Therapies: Value, Affordability, and Access. Am. J. Med. Genet. Part C Semin. Med. Genet..

[69] Berry D., Hickey C., Kahlman L., Long J., Markus C., McCombs C.K. (2025). Ensuring Patient Access to Gene Therapies for Rare Diseases: Navigating Reimbursement and Coverage Challenges. Mol. Ther. Methods Clin. Dev..

[70] Chirmule N., Feng H., Cyril E., Ghalsasi V.V., Choudhury M.C. (2024). Orphan Drug Development: Challenges, Regulation, and Success Stories. J. Biosci..

[71] Grand T.S., Ren S., Hall J., Åström D.O., Regnier S., Thokala P. (2024). Issues, Challenges and Opportunities for Economic Evaluations of Orphan Drugs in Rare Diseases: An Umbrella Review. PharmacoEconomics.

[72] Kerpel-Fronius S., Baroutsou V., Becker S., Carlesi R., Collia L., Franke-Bray B., Kleist P., Kurihara C., Laranjeira L.F., Matsuyama K. (2020). Development and Use of Gene Therapy Orphan Drugs—Ethical Needs for a Broader Cooperation Between the Pharmaceutical Industry and Society. Front. Med..

[73] Phares S., Trusheim M., Emond S.K., Pearson S.D. (2024). Managing the Challenges of Paying for Gene Therapy: Strategies for Market Action and Policy Reform.

[74] Callenbach M.H.E., Ádám L., Vreman R.A., Németh B., Kaló Z., Goettsch W.G. (2023). Reimbursement and Payment Models in Central and Eastern European as Well as Middle Eastern Countries: A Survey of Their Current Use and Future Outlook. Drug Discov. Today.

[75] Kaló Z., Niewada M., Bereczky T., Goettsch W., Vreman R.A., Xoxi E., Trusheim M., Callenbach M.H.E., Nagy L., Simoens S. (2024). Importance of Aligning the Implementation of New Payment Models for Innovative Pharmaceuticals in European Countries. Expert Rev. Pharmacoecon. Outcomes Res..

[76] Ádám I., Callenbach M., Németh B., Vreman R.A., Pontén J., Strbad T., Dawoud D., Kostyuk A., Seyam A., Nagy L. (2022). Delayed Payment Schemes in Central-Eastern Europe and Middle-East. Front. Med..

[77] Avşar T.S., Elvidge J., Hawksworth C., Kenny J., Németh B., Callenbach M., Ringkvist J., Dawoud D. (2024). Linking Reimbursement to Patient Benefits for Advanced Therapy Medicinal Products and Other High-Cost Innovations: Policy Recommendations for Outcomes-Based Agreements in Europe. Value Health.

[78] Otte M., Dauben H.P., Ahn J., Gutierrez Ibarluzea I., Drummond M., Simoens S., Kaló Z., Suh D.-C. (2024). Value Based Healthcare and Health Technology Assessment for Emerging Market Countries: Joint Efforts to Overcome Barriers. Expert Rev. Pharmacoecon. Outcomes Res..

[79] Ádám I., Callenbach M., Németh B., Vreman R.A., Tollin C., Pontén J., Dawoud D., Elvidge J., Crabb N., Van Waalwijk Van Doorn-Khosrovani S.B. (2022). Outcome-Based Reimbursement in Central-Eastern Europe and Middle-East. Front. Med..

[80] Almási T., George M., Arnaiz F., Elezbawy B., Nagy B., Kalo Z. (2020). Supporting Role of Non-Governmental Health Insurance Schemes in the Implementation of Universal Health Coverage in Developing Countries. J. Health Policy Outcomes Res..

[81] Campbell J.D., Kaló Z. (2018). Fair Global Drug Pricing. Expert Rev. Pharmacoecon. Outcomes Res..

[82] Kluszczynski T., Nemeth B., Władysiuk M., Czech M., Kamusheva M., Fotin N., Rose S., Doležal T., Hren R. (2025). Optimizing Patient Access to Orphan Medicinal Products: Lessons from Central and Eastern Europe. J. Mark. Access Health Policy.

[83] Callenbach M.H.E., Schoenmakers D., Vreman R.A., Vijgen S., Timmers L., Hollak C.E.M., Mantel-Teeuwisse A.K., Goettsch W.G. (2024). Illustrating the Financial Consequences of Outcome-Based Payment Models from a Payers Perspective: The Case of Autologous Gene Therapy Atidarsagene Autotemcel (Libmeldy^®^). Value Health.

[84] Gentilini A., Neez E., Wong-Rieger D. (2025). Rare Disease Policy in High-Income Countries: An Overview of Achievements, Challenges, and Solutions. Value Health.

[85] Brooks P.J., Miller T.M., Revah F., Suh J., Garrison B.R., Starke L.C., MacLachlan T.K., Neilan E.G., Raychaudhuri G., Kassim S.H. (2024). The Bespoke Gene Therapy Consortium: Facilitating Development of AAV Gene Therapies for Rare Diseases. Nat. Rev. Drug Discov..

[86] Voelker R. (2021). Public-Private Partnership Sets Its Sights on New Gene Therapies. JAMA.

[87] Hren R., Dóczi T., Orszagh E., Babič D. (2025). Recent Advances in Perfusion Assessment in Clinical Oncology Using Hyperspectral Imaging. Electronics.

[88] Hren R., Dóczi T., Országh E., Kocjan T. (2026). The Significance of Hypophosphatemia in Deciding on an Optimal Clinical Choice of Parenteral Iron Therapy in Patients with Chronic Inflammatory Bowel Disease in Slovenia: An Umbrella Review and Economic Evaluation. Healthcare.

[89] Kovács S., Kaló Z., Daubner-Bendes R., Kolasa K., Hren R., Tesar T., Reckers-Droog V., Brouwer W., Federici C., Drummond M. (2022). Implementation of Coverage with Evidence Development Schemes for Medical Devices: A Decision Tool for Late Technology Adopter Countries. Health Econ..

[90] Imre A., Balogh B., Mándity I. (2024). GraphCPP: The New State-of-the-Art Method for Cell-Penetrating Peptide Prediction via Graph Neural Networks. Br. J. Pharmacol..

[91] Imre A., Dombi G., Dobó M., Mhammad A., Ferencz E., Balogh B., Vincze A., Szabó Z.-I., Balogh G.T., Rácz A. (2025). Machine Learning-Assisted Retention Time Predictions on a Cellulose Tris(3,5)-Dimethylphenylcarbamate Column in Polar Organic Mode. Anal. Chim. Acta.

[92] Erdei E., Deme R., Balogh B., Mándity I.M. (2025). Cell-Mediated and Peptide-Based Delivery Systems: Emerging Frontiers in Targeted Therapeutics. Pharmaceutics.

[93] Lane J., Edwards R.T., Babarczy B., Whiteley H., Oruganti V., Rutten-van Mölken M., Costongs C., Jani A.R., Wordsworth S., Maassen A. (2025). A Protocol for Mobilising Novel Finance Models for Collaborative Health Promotion and Disease Prevention Initiatives: Taking a Smart Capacitating Investment Approach in the Invest4Health Project. Front. Public Health.

[94] Jakab I., Dimitrova M., Houÿez F., Bereczky T., Fövényes M., Maravic Z., Belina I., Andriciuc C., Tóth K., Piniazhko O. (2023). Recommendations for Patient Involvement in Health Technology Assessment in Central and Eastern European Countries. Front. Public Health.

[95] Murányi M., Hosszú D., Rózsa P., Szegner P., Koltai T., Kovács V., Lovas K., Pogány G., Tóth K., Szécsényi-Nagy B. (2025). Defining the Concept and Potential Roles of Patient Organizations in Hungary. Orvosi Hetil..

[96] Damodaran A. Cost of Capital (WACC) Data 2025. https://pages.stern.nyu.edu/~adamodar/New_Home_Page/datafile/wacc.html.

[97] Lapteva L., Purohit-Sheth T., Serabian M., Puri R.K. (2020). Clinical Development of Gene Therapies: The First Three Decades and Counting. Mol. Ther.-Methods Clin. Dev..

[98] Khan G., Hashim M.J. (2025). Epidemiology of Multiple Sclerosis: Global, Regional, National and Sub-National-Level Estimates and Future Projections. J. Epidemiol. Glob. Health.

[99] Murthy N., Purwar S., Sarshad M., Leggett I. (2018). To Spend or Not to Spend: Investing for Launch Success as an Emerging Company. https://www.syneoshealth.com/insights-hub/spend-or-not-spend-investing-launch-success-emerging-company.

